# Karst Detection Beneath the Pyramid of El Castillo, Chichen Itza, Mexico, by Non-Invasive ERT-3D Methods

**DOI:** 10.1038/s41598-018-33888-9

**Published:** 2018-10-18

**Authors:** René E. Chávez, Andrés Tejero-Andrade, Gerardo Cifuentes, Denisse L. Argote-Espino, Esteban Hernández-Quintero

**Affiliations:** 1 Instituto de Geofísica, Universidad Nacional Autónoma de México, Cd. Universitaria, Circuito Exterior s/n, 14260 Mexico City, Mexico; 2 Facultad de Ingeniería UNAM, Cd. Universitaria, Circuito Interior s/n, 14260 Mexico City, Mexico; 3 Dirección de Estudios Arqueológicos, Instituto Nacional de Antropología e Historia. Lic. Primo Verdad no. 3, Col. Centro, Deleg, Cuauhtémoc, CDMX Mexico

## Abstract

Currently, archaeologists perform excavations determined by previous geophysical studies to accurately establish the prospective targets and minimize site disturbance. Among others, one of the methods most widely employed is the Electrical Resistivity Tomography (ERT-2D, -3D). However, investigation of the subsoil of archaeological buildings is not possible to carry out with traditional geophysical methods, because the structure itself prevents it. Therefore, it is necessary to design non-invasive special arrays capable of characterizing the subsoil of such buildings, while preserving their historical context. Here we show how this procedure combined with sequences of resistivity observations at depth allowed us to detect a low resistivity body beneath the pyramid of *El Castillo* in Chichen Itza (Mexico). This feature may be associated with a cavity (karst) partially filled with sweet water. On the other hand, a natural cavity was discovered under *El Osario* pyramid (south of El Castillo), at the end of the 19th century. Therefore, this pyramid was also studied to validate the effectiveness of this methodology, obtaining outstanding results. This method provides an interesting procedure to investigate the subsoil of archaeological structures for unveiling evidences that allow specialists to understand the religious meaning of these temples.

## Introduction

The ERT method has been applied to solve a wide variety of problems mainly focused on detecting buried structures in different areas of application^1,2^. Conventional ERT-3D surveys have been employed in archaeology with successful results^3,4^. However, this method is challenged, when it is necessary to characterize the subsoil beneath historical monuments in archaeological sites. Such ‘obstacles’ prevent the deployment of parallel profiles to form grids^5,6^. Furthermore, drilling to insert the electrodes into the ground is forbidden in order to preserve intact the historical context of the site. A non-invasive methodology was designed to overcome this problem^7,8^, where different electrical arrays are developed to characterize the subsoil beneath the archaeological structures, centered on traditional ERT-3D arrays enclosing in a square geometry the base of the studied edifice.

**Figure 1:**
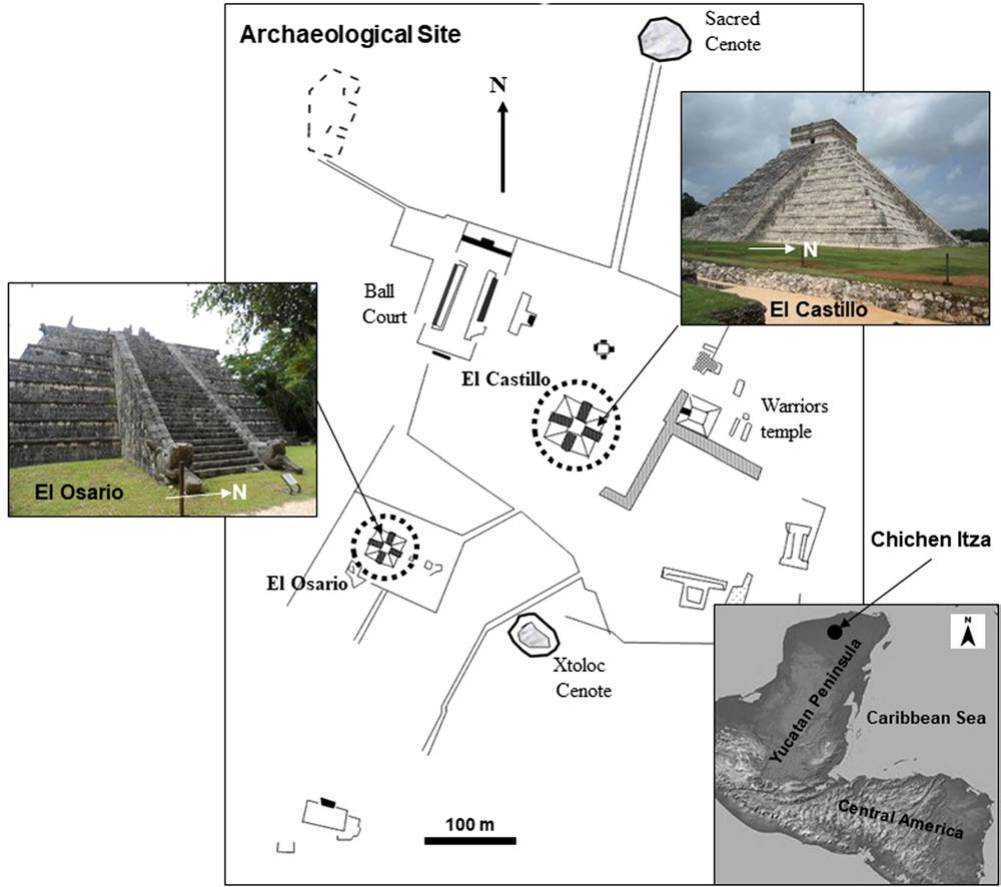
The archaeological site of Chichen Itza, Yucatan, Mexico is shown in this map. The Pyramid of *El Castillo* (solid circle and top-right inset) and *El Osario* (broken circle and left inset) are depicted, as well as other important Mayan edifices and constructions. The solid circle depicts the location of this site within the Peninsula of Yucatan (lower inset).

This procedure was suitable for investigating pre-Hispanic monuments like the pyramid of *El Castillo* (Fig. 1, top-right inset and broken circle). This temple is located within the ancient Mayan city of *Chichen Itza* (Fig. 1), and is considered the most emblematic pyramid of this ancient civilization, elected as one of the seven man-made world wonders and part of Mankind’s Heritage. The last stage of *El Castillo* was probably built between 900–1000 A.D^9^. It is composed of 9 bodies, a temple at the top of the pyramid and four staircases with 91 steps each (including the platform at the top, adding up to 365 steps, the number of days in our solar year)^10^.

Chichen Itza is one of the major Mesoamerican cities, established in an area of 20 km^2^ in the southern lowlands of Mexico, within the Yucatan Peninsula (Fig. 1, bottom-right inset). Sinkholes and cavities carved in the limestone rocks at depth are found within the ancient city limits. Some of them were devoted to religious ceremonies and provided of fresh water to the ancient Mayas^9^. Specialists^10–12^ believe that the city was occupied at different times by diverse ethnic groups. Although settlements around the site can be dated back to the Late Preclassic period (500 B.C.). A major development of the city began in the Late Classic period (after 600 A.D.), reaching its peak with an intensive monumental activity stage between 900 and 1100 A.D. Finally, from 1100 to 1300 A.D. became the decadence of the civilization. The reconstruction of the historical evolution of this important city is based on architectural styles, ceramic types and thermoluminescence methods^11–13^.

Previous Ground Penetrating Radar (GPR) studies carried out within the pyramid’s main plaza^14^, provided evidence of a buried trench excavated within the limestone rock and hidden under infill materials. Such infill covers the platform of the main plaza, which probably corresponded to the ‘*Great Leveling*’ carried out during the last constructive period^10,11^. The inferred feature seemed to disappear under the eastern stairway of the Pyramid of *El Castillo*^15^.

On the other hand, *El Osario* was built around 980 A.D. as a smaller version of the pyramid of *El Castillo*^16^. This structure is located 500 m southwest of this iconic edifice (Fig. 1, broken circle and left inset), and near the Xtoloc Cenote. E. Thompson^17^ unveiled in 1897 a vertical shaft entrance at the top of *El Osario* pyramid. This stone-lined shaft descended vertically to the base of the pyramid, entering 12 m deep into a natural cave, where seven burials were discovered along with several objects made of jade, copper bells, rock crystal and shell^16,17^. Similarities in architectural styles between this pyramid and *El Castillo*, made the archaeologists^16,18^ believe that similar conditions could exist beneath this structure.

Therefore, the present investigation is focused on exploring *El Castillo* pyramid’s subsoil by employing the ERT-3D method, using unconventional and non-invasive geoelectric arrays.

## Results

The pyramid of *El Osario* was initially studied to demonstrate the effectiveness of the proposed ERT-3D method, by employing unconventional and non-invasive geoelectric arrays^7,8^. As mentioned above, this structure was built on top of a natural cavity, which was discovered in the late 19th century^17^. The results obtained are highly relevant to investigate the pyramid of *El Castillo* subsurface, which might present the same characteristics of *El Osario*^18^.

### El osario pyramid

**Figure 2:**
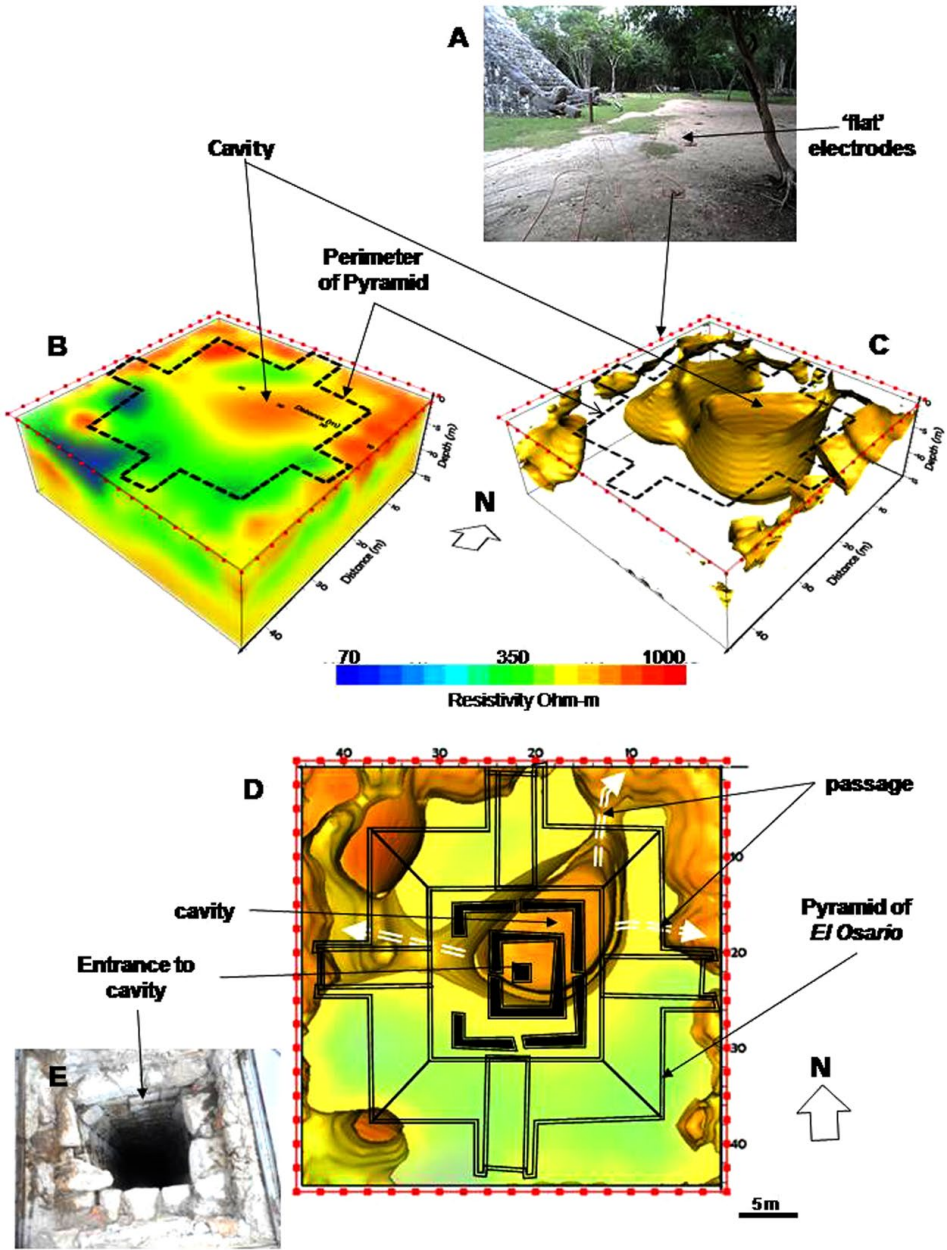
To demonstrate the possibilities of the proposed ERT-3D method, 72 electrodes were deployed around the base of the temple of *El Osario* (**A**). The inverted model shows a high resistivity anomaly towards the central part of the WRC (**B**). This anomaly can be isolated and a void structure can be observed, corresponding to the mentioned cavity^17^ (**C**). The image of the WRC, seen from above, defines the dimensions of the structure, and the 3 passages that connect it (**D**, broken arrows). The outline of *El Osario* was placed on top of the resistivity model for a better visualization, as well as the location of the vertical shaft entrance^17,18^ (**E**).

A series of flat-base cooper electrodes were deployed around the base of the pyramid (Fig. 2A) to test the ERT-3D methodology proposed. A special sequence of resistivity observations was acquired to study the subsoil of the pyramid by employing the ERT-3D method.

The inverted resistivity data from the Pyramid of *El Osario* is displayed in Fig. 2B. The Working Resistivity Cube was obtained after 7 iterations with a RMS = 4%^19^, where a smooth inverted resistivity distribution at depth was achieved. A lateral view of the Working Resistivity Cube is shown, where several prominent features can be observed. The approximate perimeter of the pyramid has been outlined to facilitate the correlation of the detected anomalies (broken line).

An elongated anomaly (~1000 Ohms-m) can be observed extending approximately towards the central portion of the Working Resistivity Cube. This feature possesses an interesting geometry that stands out above the rest of the geoelectric anomalies. Other high resistivity anomalies could be associated to the pyramid foundations, infill materials employed to level the terrain or voids in the limestone. Resistivity values about the order of 350 Ohm-m could be related to the natural rock in the area, limestone in this case. Lower resistivity values (~70 Ohm-m) may represent water-saturated areas of seeping through the pyramid into the ground due to rainwater.

A range of resistivity values (300 Ohm-m to 500 Ohm-m) were considered and displayed as iso-surfaces to obtain the image in Fig. 2C. The morphology of the already mentioned high resistivity anomaly can be observed more clearly. This feature is defined by a hollow structure, whose wrapping represents the surrounding medium (limestone), and reaches an approximate depth of 14 m from the base of the pyramid to its lowest portion. This natural structure must be the resistivity signature of the cavity already reported E. Thompson^17,18^.

The pyramid’s outline is shown on top of the Working Resistivity Cube (Fig. 2D). This image shows the resistivity model seen from the top of the pyramid. The inferred cavity possesses an approximate diameter of 15 m. Today’s entrance to the vertical shaft^17^ is shown in image (2E). Observe that such feature is not precisely centered on the midpoint of the cavity^18^. On the other hand, 12 different exits were reported inside the cave^17^, overlooking their course. Our ERT-3D study could only depict three different passages displayed in Fig. 2D (broken arrows). One inferred channel run towards the northwestern direction, where the Pyramid of *El Castillo* is found. This model leads us to consider a possible underground connection between *El Osario* and *El Castillo* pyramids. It will be necessary to perform in the future further ERT-2D lines in between the two pyramids to demonstrate it. Other passage runs in the western direction towards the Cenote Xtoloc, partially explored by E. Thompson^17^, and the third one to the east.

### El castillo pyramid

**Figure 3:**
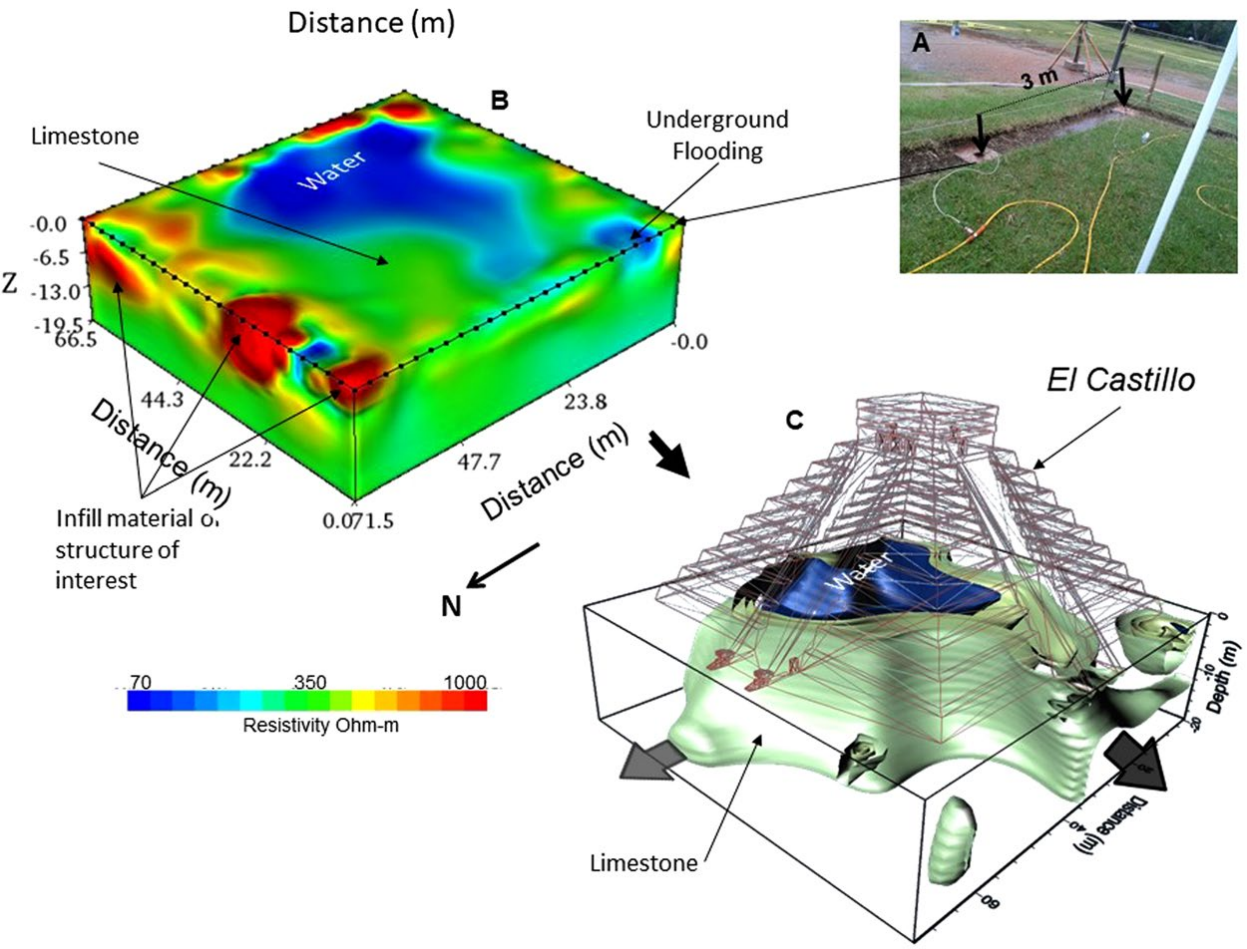
The flat-base electrodes were placed in a trench around the base of the Pyramid of El Castillo, separated 3 m (**A**). The WRC shows a water-saturated zone towards the center of the pyramid (~70 Ohm-m) (**B**). It is possible to isolate that resistive structure. The geometry of a cavity partially filled with water is observed, where the digital image of the pyramid has been placed on top of the WRC for a better visualization of the structure (**C**).

The famous pyramid of *El Castillo* subsoil was investigated by deploying around its base 96 flat-base electrodes, separated 3 m (Fig. 3A). 5 iterations were needed with a RMS error of 2.1%^19^ to obtain the inverted resistivity model displayed in Fig. 2B. Low resistivity values (<80 Ohm-m) revealed evidence of a body saturated with sweet water beneath the Pyramid of *El Castillo* extending 20 m in the N-S direction and 16 m in the E-W direction, approximately. Intermediate resistivity values (~350 Ohm-m) could be associated with the limestone rocks. High resistivity values (>800 Ohm-m) might correspond to materials employed in the different constructive periods of the pyramid.

When displaying only low resistive values (~80 Ohm-m) as iso-surfaces, the geometry associated to the highly saturated materials is clearly observed (Fig. 2C). The feature surrounding the low resistivity body might correspond to a drier feature (limestone, ~350 Ohm-m), suggesting the geometry of a buried cavity (or karst) partially filled with water. For best viewing, we have placed the digital image of the pyramid on top of the Working Resistivity Cube. The arrows in the figure attempt to represent the possible direction of the groundwater flow. This interpreted cavity (karst) extends beneath the pyramid from the surface to more than 20 m deep.

Under the present results, several questions arise, which might be answered by the specialists on the Mayan culture:

Did the ancient Mayans know about the existence of this subterranean structure? If so, why did the Mayans built this huge temple on top of this structure?

Further archaeological and geophysical investigations will provide the answers to such questions.

### Synthetic modelling

It is important to point out that the inverted resistivity model obtained for the pyramid of *El Castillo* shows an open cavity towards the surface, as if most of this emblematic edifice was built on that ‘open surface’. Such a result means that the method employed depicts a poor vertical resolution towards the center of the ERT array due to the lack of resistivity observations on this portion, forcing the inversion to pop out the body on to the surface in the inversion process^8,20^. A similar inaccuracy was observed for the inverted resistivity model of *El Osario*, where the inferred cavity outcrops on the surface beneath the base of the pyramid. However, E. Thompson^17^ discovered the entrance to this cavity 3 m below the base of the pyramid. Such lack of vertical resolution is due to the inversion algorithm used, which is part of the commercial software employed to carry out the inversion process. Some authors^8,20^ have mentioned that such commercial algorithms, often considers grids formed by lines of electrodes to deal with a 3D interpretation. However, the methodology exposed in this investigation only needs electrode lines surrounding the studied targets^7,8,20^. Then, it is crucial to analyze the effectiveness of the methodology employed in order to understand the results obtained for *El Osario* and *El Castillo* pyramids. Therefore, synthetic examples have been designed to examine the behavior of the inverted solutions and to consider the possibilities and limitations of the arrays applied. Two different models have been designed to analyze the inversion results.

### Synthetic model 1

**Figure 4:**
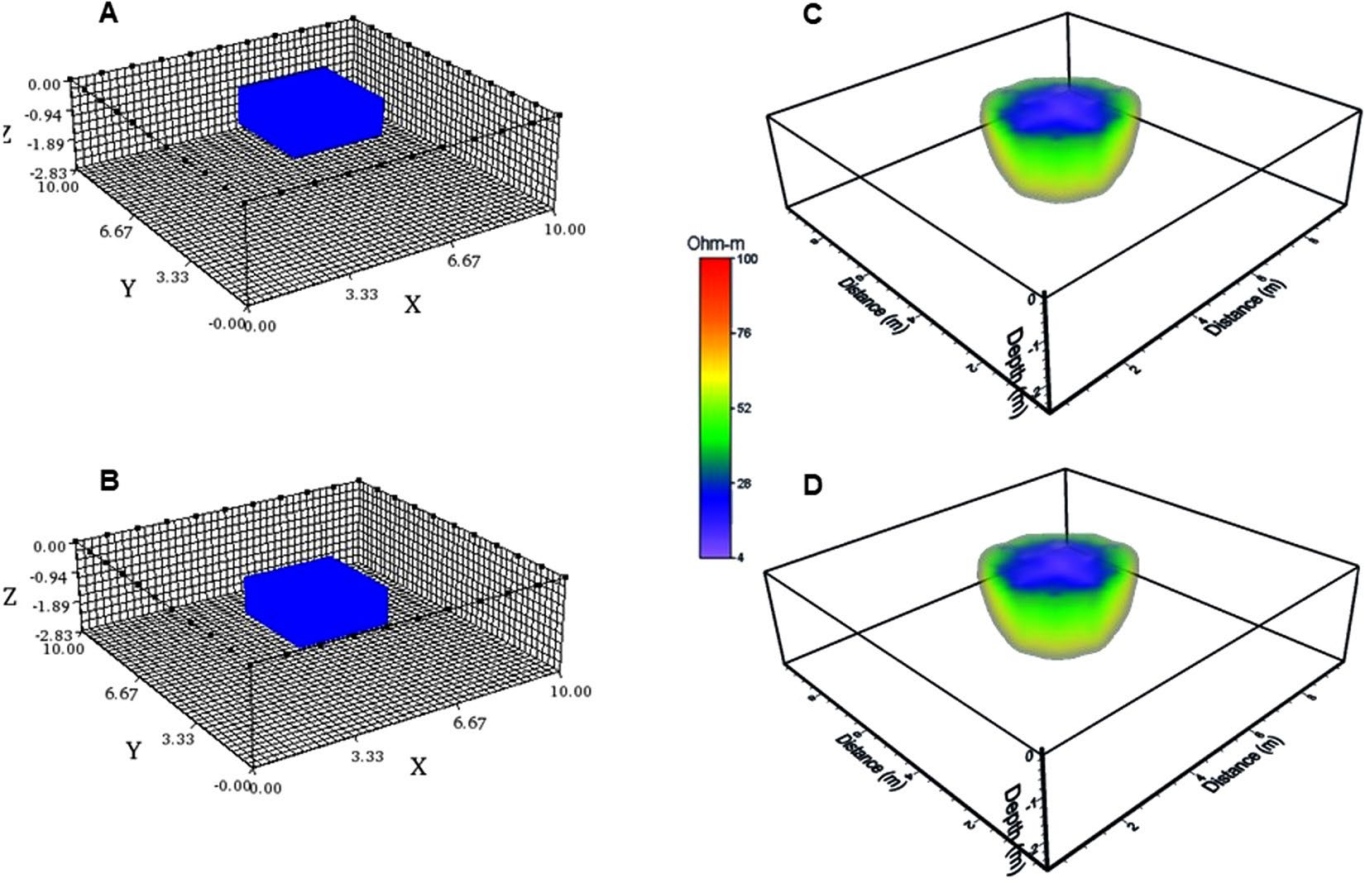
A synthetic model cube is computed, located towards the central portion of a 3D grid of 10 × 10 m^2^. Initially, the block outcrops (**A**) and then the model is buried 2 m from its top (**B**). Inverted solutions obtained are quite similar (**C**,**D**). The inverted solutions pop up to the surface, indicating a poor vertical resolution of the arrays employed.

Initially, we have considered an array comprised by 40 electrodes separated 1 m, deployed in a square geometry. A grid of 10 × 10 m^2^ was constructed to model the response of the resistive synthetic model. Such model is represented by a block of 4 × 4 × 2 m^3^, and positioned towards the central portion of the grid. The resistivity value assigned to the model was 10 Ohm-m embedded in a 100 Ohm-m half-space. The ERT-3D arrays have been included to compute the resistivity response of this block. Two different cases are dealt with, first the model outcrops to the surface (Fig. 4A), and second, the model is buried at a depth of 2 m (Fig. 4B).

It is important to point out that the inverted models look quite similar (Fig. 4C,D). In both diagrams, the inversion algorithm inserted in the commercial program EarthImager3D^19^, vertically prolongs the solution towards the surface for the buried block, nevertheless the horizontal resolution is adequate and its original lateral dimensions have been recovered as well as its resistivity value. Unfortunately, the lack of data towards the central portion of the block cannot resolve the actual depth of each block.

### Synthetic model 2

**Figure 5:**
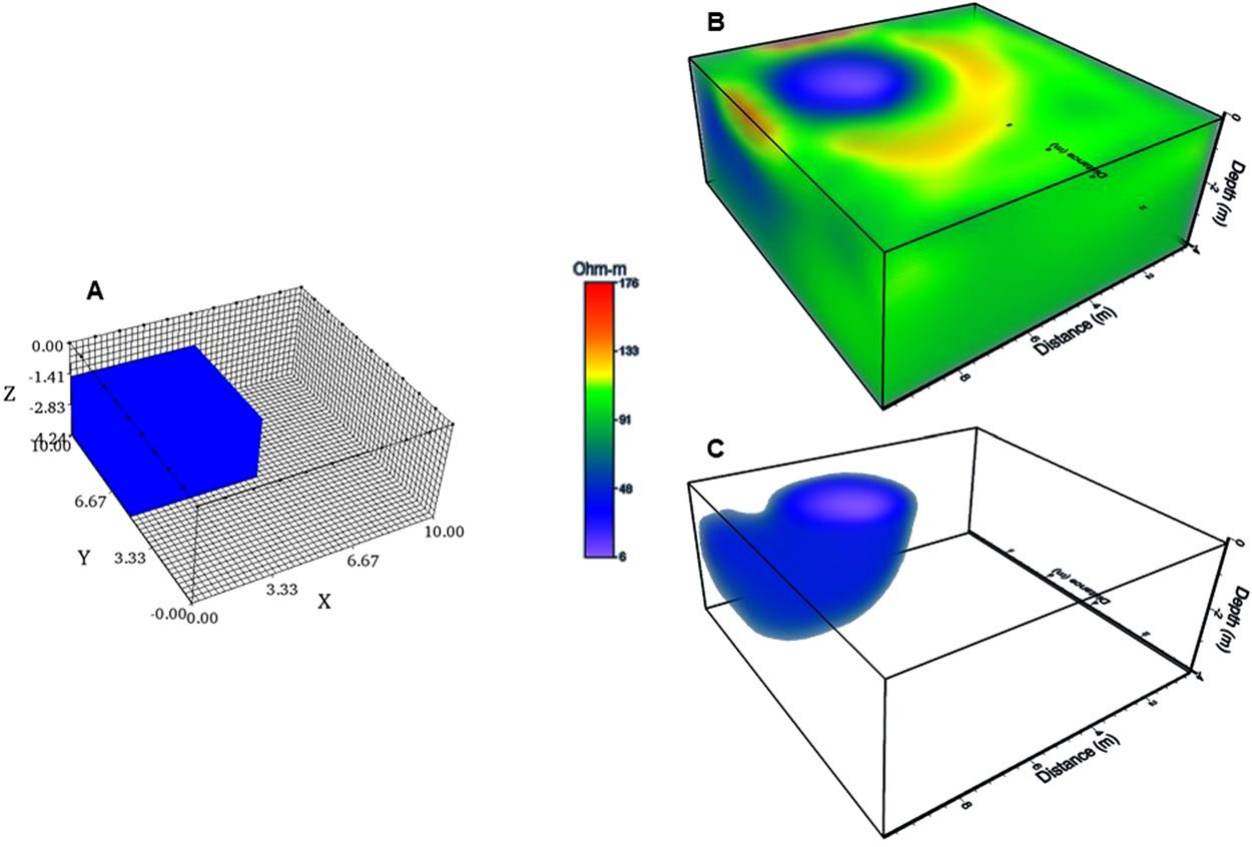
A synthetic cube is shown positioned towards the upper left-hand corner of the 10 × 10 m^2^ grid (**A**). The inverted solution also is stretched unto the surface (**B**). Plotting the interval between 1 Ohm-m to 10 Ohm-m (**C**), the geometry of the inverted block is depicted distorted and its top outcrops. However, the lateral dimensions and the original resistivity of the block is well recovered.

Let us now consider a block model located towards the left-upper corner of the Working Resistivity Cube (Fig. 5A). The dimensions of this block are: 5 × 5 × 2.5 m^3^, with an assigned resistivity of 10 Ohm-m, embedded as the previous example in a 100 Ohm-m half-space. The grid is 10 × 10 m^2^ as well, with 40 electrodes separated 1 m, forming a square geometry. The three arrays discussed in this investigation have also been applied to calculate the resistivity response. It is important to say that the observed data has been contaminated with noise (5%).

The inverted solution (Fig. 5B) depicts a circular low resistivity anomaly on the surface, within the interval of 10 Ohm-m. The green color corresponds to the half-space with a resistivity of 91 Ohm-m. High resistivity values surrounding the main anomaly may correspond to noisy effects. The program allows removing not required data, leaving values within an interval of 1 Ohm-m to 10 Ohm-m. Figure 5C depicts the isolated inverted structure. The correct geometry of the original synthetic model has not been recovered; nevertheless, position, lateral dimensions and its resistivity value are well defined. As in the previous example, the inverted solution depicts an outcropping block and again, a lack of vertical resolution.

It has been shown that the arrays employed present a poor vertical resolution, because the inversion algorithm employed expects to have data on top of the anomalous resistivity structure^8,20^, which is not the case in the methodology presented. We must say that there is not a physical or theoretical restriction on the way to display the electrodes^20^, as well as the geometry designed to deploy them^8^. This means that the ERT-3D arrays treated here, will present such limitations, when applied on real data; nevertheless, lateral dimensions of the synthetic models presented are well achieved. Therefore, we can conclude that the resistivity models obtained for the pyramids of *El Osario* and *El Castillo* possess a good estimation on their lateral distribution and do not have an adequate vertical resolution.

## Discussion

The characteristics of the ERT-3D method and its flexibility made it possible to acquire sufficient resistivity observations to obtain a reliable subsurface resistivity model. In addition, this method does not disturb the archaeological context, especially by employing the flat-base electrodes. However, the modality employed in this research presents the same problems that traditional electrical methods possess. Such as the geological conditions of the terrain (humidity, materials with high resistivity and so on), which increase the error in the data acquired and decrease the depth of investigation.

The resistivity models obtained present a good lateral response; nevertheless, they offer a poor vertical resolution, as shown by the synthetic models treated. The inversion algorithm employed expects to have data on top of the anomalous resistivity structure, as discussed by different authors^8,20^. This is a limitation in current commercial softwares, which are not prepared to handle ERT-3D arrays, like the ones applied in this investigation. Therefore, it will be necessary to work on an inversion algorithm in the future, that includes enhancement techniques that could help to improve the vertical resolution of an inverted resistivity model, introducing global optimization inversion methods^20^.

The methodology discussed confirms the presence of a natural cave beneath *El Osario* pyramid, discovered in 1897^17^, within the limitations commented earlier. The interpreted resistivity model for the pyramid of *El Castillo* subsoil, suggests the existence of a cavity (karst) carved in the limestone rock beneath the temple, extending to a depth of 20 m, and partially filled with sweet water, and open wide towards the surface. It was mentioned that this is due to the lack of observation on the surface central portion of the array.

Caves for the ancient Mayans were symbolic places, where opposing forces, material and supernatural, interacted^21^. These were also sources of water and construction supplies^9,10^. Therefore, the ancient settlers of *Chichén Itzá* probably knew the existence of this feature (karst) and selected this precise spot to build their main temple. This hypothesis is supported by the discovery of a previous constructive phases inside *El Castillo* pyramid^18^, centered on top of this feature^20^. These results may lead to search for significant archaeological information about this ancient civilization, which could be hidden within this geological feature.

## Methodology

ERT-3D data were obtained employing a *Syscal-Pro Resistivimeter*^22^ with 48 channels connected to a switch box that allowed extending to 96 channels, interconnected to the main console with 4 sets of cables of 120 m of length each. Traditional electrodes (copper-made poles) could not be inserted into the soil of the area of study due to the existence of ancient Mayan floors and structures within the Main Plaza of *Chichen Itza*, recently discovered by archaeological excavations carried out near the pyramid of *El Castillo*. Therefore, special flat-base electrodes were designed, consisting in a square cooper-made plate with a flap at the center, with an area of 0.25 × 0.25 m^2^ and a thickness of 0.3 cm. Special flat-base detectors have been employed before in urban and archaeological sites^23,24^ with successful results. A water-soluble gel (usually employed in ultrasound medical analysis) was applied to the contact surface of each plate to ensure a good transmission of the electrical current from the electrodes to the subsoil.

Sometimes, spatial conditions of the surveyed area do not allow deployment of conventional ERT-3D settings. Such constraints are imposed by natural or anthropogenic ‘*obstacles*’, which are often the targets of the proposed survey. Alternative arrays must be developed to solve this problem, employing non-invasive geometries to characterize the subsoil beneath such archaeological structures.

**Figure 6:**
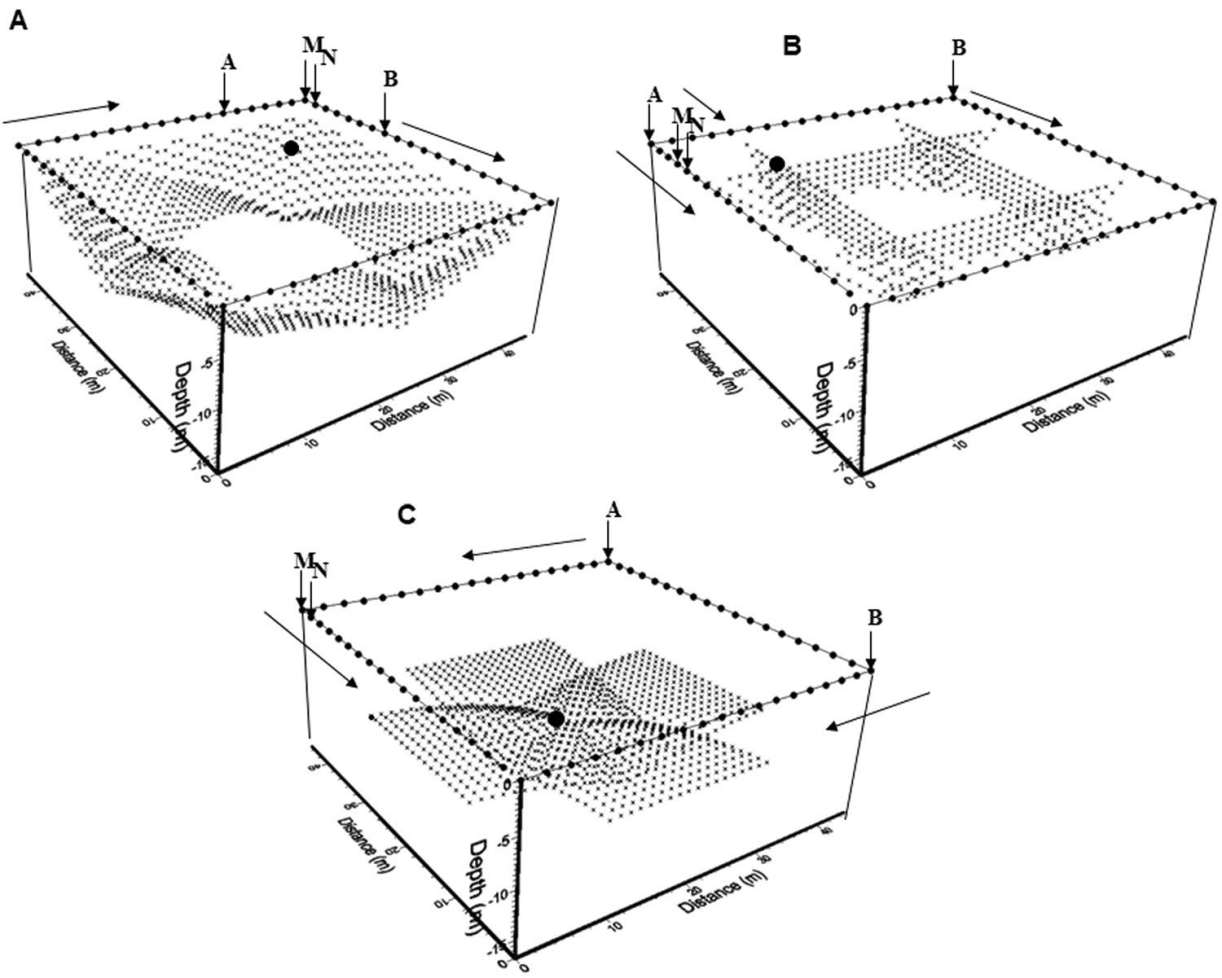
The methodology to acquire the apparent resistivity data (solid circles) are depicted for the WSP (**A**), the MC (**B**) and the WSG (**C**) arrays. The distribution of the flat-base electrodes (solid circles) conforming a square geometry is depicted. The AB are the current electrodes and MN are the potential electrodes. Arrows indicate the direction of the data acquisition process and the big solid circle indicates the resistivity value obtained with the electrode array ABMN displayed in the diagrams.

Three different types of arrays were employed to investigate the pyramids of, *El Osario* and *El Castillo* (Fig. 6, electrodes are shown as solid circles). The Wenner-Schlumberger-perimeter^8^ (Fig. 6A: WSP), the Minimum Coupling^8^ (Fig. 6B: MC) and the Wenner-Schlumberger-Gradient^20^ (Fig. 6C: WSG). These electrical settings enclose into a square the base of the structure under study. The process to compute the apparent resistivity observations from these arrays are described in detail in previous publications^7,8,20^. However, a brief description of these three settings employed in this investigation is made. Note, that the arrows in Fig. 6 indicate the direction of the data acquisition.

The WSP array is initially programmed as a common two-dimensional Wenner-Schlumberger array, where **A** and **B** are the current electrodes and **M** and **N** are the potential electrodes (Fig. 6A). The set of **ABMN** electrodes move in the clockwise direction following the square geometry. When the circuit is completed, the first level of observation is obtained. Next, the distance between **AM** and **NB** is sequentially increased by one electrode spacing, keeping the **MN** separation constant, and again, when the circuit is completed, a second level of observation is registered. This process is repeated until the electrodes cover fully the square geometry and the last observational level is computed. Figure 6A shows a given position of the set of electrodes **AMNB** producing the observation point marked with a black circle. It is important to say, that such an array generates a series of observations towards the interior of the apparent resistivity cube, when the electrode setting is located on each edge of the cube. On the other hand, it is important to point out, that there is a lack of information towards the central portion of the working cube.

The MC array requires two parallel lines of electrodes, where **A** and **B** are positioned at the beginning of each line. The potential electrodes **MN** are kept fixed at a constant distance on any electrode line. Such electrodes are moved in the direction of the arrow. When the potential electrodes reach the end of the line, the first level of observation is measured. Then, the current electrodes **AB** move one electrode distance (in the arrow direction) and the **MN** electrodes move back one electrode separation from electrode **A** and sequence starts again. When the **MN** electrodes reach the end of the line, a second level of observation is completed. The process is repeated until that line is fully covered. Observe that this process builds up a TRE-2D profile of apparent resistivity observed data inside the working cube (Fig. 6B). The next step will be to set the **MN** electrodes in the next parallel line and repeating the process again to obtain the following TRE-2D profile. This process is repeated for the remaining perpendicular lines of the cube, setting the **AB** electrodes at the beginning of these lines. The complete sequence of observations constructs four TRE-2D profiles inside the working cube, as Fig. 6B depicts. This setting partially covers shallower and deeper regions of the working resistivity cube, however, as mentioned before, the central portion of the cube is not. A similar process was already reported^25^ for outlining near-surface buried structures, employing only two lines of electrodes.

The WSG array was designed to obtain information towards the deep central portion of the resistivity cube. The sequences of observations are constructed by setting initially the current electrodes **AB** in any of the two corners of the cube, then the potential electrodes **MN** are placed at the beginning of the opposite line of electrodes (Fig. 6C). Then, the observation process starts by moving the potential electrodes in the direction of the arrow, while the current electrodes remain in a fixed position. The first level of observation ends when the potential electrodes **MN** reach the last point in that line. The next sequence starts by moving the current electrodes one electrode distance in the arrow direction. Again, the **MN** move on the same line as before. The complete sequence is ended, when the **AB** electrodes reach the position before the last point on its line. As mentioned above, the sequence of observations of apparent resistivity covers the deepest central portion of the cube quite well, however, the shallow central part does not possess information.

The three sequences described are programmed in the resistivity-meter (Syscal-Pro), in such a way that the instrument automatically acquires the resistivity data. The depth of the attribution point was calculated as the median value, where 50% of the current flows above the attribution point and the other 50% flows beneath it^8,26,27^

When the data acquisition ends, the resulting apparent resistivity data can be downloaded into a PC and merged into a single data file ready to be inverted to obtain the real resistivity values. Data inversion is based on the method^28^ programmed in the commercial software EarthImager 3D^19^ used in this investigation to calculate the real resistivity distribution at depth. The objective function **S**(**m**) of a smooth model inversion, utilizing the norm L2, is expressed as^28,29^:

$${\bf{S}}({\rm{m}})={({{\bf{d}}}_{{\rm{obs}}}-{\rm{g}}({\bf{m}}))}^{T}{{\bf{W}}}_{{\boldsymbol{d}}}({{\bf{d}}}_{{\boldsymbol{obs}}}-{\bf{g}}({\bf{m}}))+\propto \cdot {{\bf{m}}}^{T}{\bf{Rm}}$$

where **m** is the vector of model parameters. It is possible to select, during the inverse process, parameters α (the smoothness factor) and the damping factor, which is part of the weighting matrix **W**_***d***_. These values determine the amount of model roughness (**R**) during the inversion process. The difference between the observed (**d**_***obs***_) and computed (**g**(**m**)) data is obtained. In our case, we have used the following values for α = 10, the damping factor = 10, the minimum resistivity = 100 Ohm-m, and the maximum resistivity = 1000 Ohm-m.

**Figure 7:**
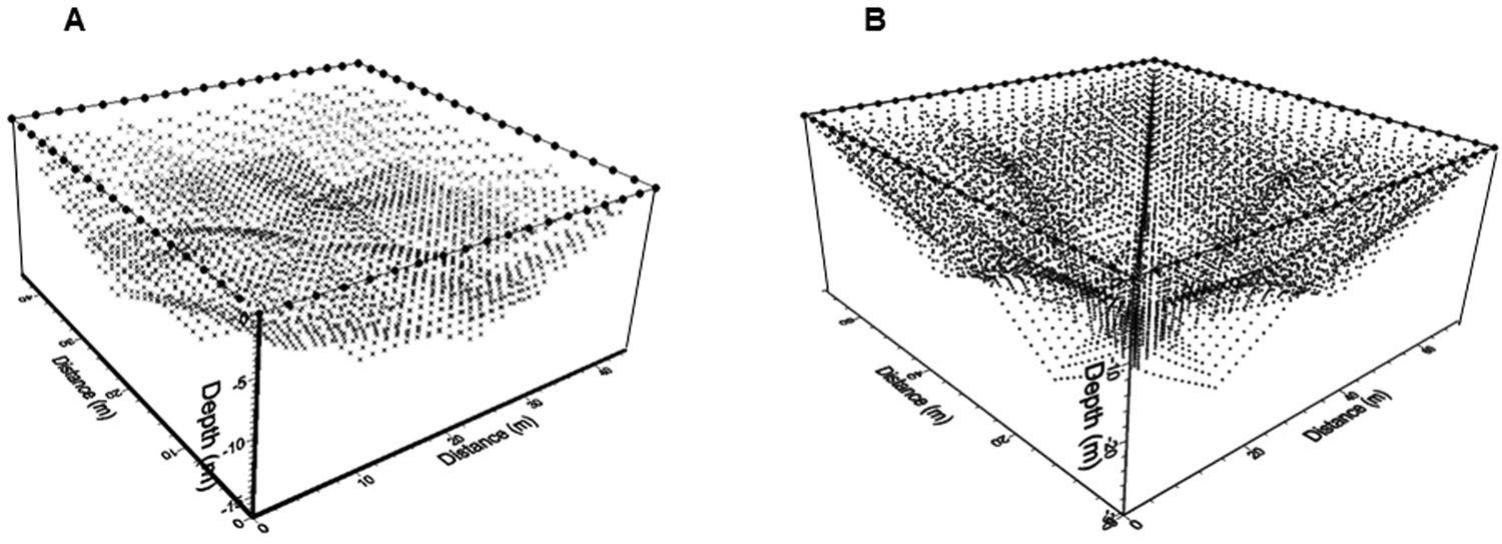
Combining the arrays discussed in the text, the total of 5200 resistivity data to be collected for the Pyramid of *El Osario* is shown (**A**). The temple of *El Castillo* subsoil required 7192 observations (**B**).

These arrays were employed to characterize *El Osario* and El Castillo pyramids subsoil. The combined sequences provided the apparent resistivity observations measured at depth. A series of 5200 observations at depth were acquired at varying depths for the temple of *El Osario* (Fig. 7A). The theoretical depth^8^ reached by this geometry was 17 m, with an interval separation between electrodes of 2.5 m, employing a total of 72 copper flat-base electrodes.

The pyramid of *El Castillo* is a much larger structure, possessing an area of 66.5 × 71.5 m^2^. Therefore, we deployed 96 cooper flat-base plates surrounding this magnificent edifice, with an electrode separation of 3 m. These electrodes were placed along a small trench of 0.30 m wide and 0.05 m deep (Fig. 3A), where the grass was removed, so that the flat surface of the electrodes made direct contact with the natural soil. A sequence of 7192 apparent resistivity data (Fig. 7B) was acquired, with a theoretical depth of investigation^8^ of 25 m.

As mentioned before, the sequences and the geometric distribution of these observations are downloaded in the resistivimeter computer memory for the acquisition process. It is important to quote that there is a lack of information towards the central shallow portion of the Working Resistivity Cube (Fig. 7), nevertheless, a good coverage at depth is obtained for both pyramids by adding the three arrays employed in this investigation.
